# *RET* Fusion Testing in Patients With NSCLC: The RETING Study

**DOI:** 10.1016/j.jtocrr.2024.100653

**Published:** 2024-02-20

**Authors:** Esther Conde, Susana Hernandez, Jose Luis Rodriguez Carrillo, Rebeca Martinez, Marta Alonso, Daniel Curto, Beatriz Jimenez, Alejandra Caminoa, Amparo Benito, Pilar Garrido, Sergi Clave, Edurne Arriola, Isabel Esteban-Rodriguez, Javier De Castro, Irene Sansano, Enriqueta Felip, Federico Rojo, Manuel Dómine, Ihab Abdulkader, Jorge Garcia-Gonzalez, Cristina Teixido, Noemi Reguart, Desamparados Compañ, Amelia Insa, Nuria Mancheño, Sarai Palanca, Oscar Juan-Vidal, Nuria Baixeras, Ernest Nadal, Maria Cebollero, Antonio Calles, Paloma Martin, Clara Salas, Mariano Provencio, Ignacio Aranda, Bartomeu Massuti, Laura Lopez-Vilaro, Margarita Majem, Luis Paz-Ares, Fernando Lopez-Rios

**Affiliations:** a Hospital Universitario 12 de Octubre, Madrid, Spain; b Universidad Complutense, Madrid, Spain; c Research Institute Hospital 12 de Octubre (i+12), Madrid, Spain; d Centro de Investigación Biomedica en Red Cancer (CIBERONC), Madrid, Spain; e Hospital Universitario Infanta Sofía, Madrid, Spain; f Hospital Quirón Salud, Madrid, Spain; g Hospital Universitario Fuenlabrada, Madrid, Spain; h Hospital Universitario Ramon y Cajal, Madrid, Spain; i Hospital del Mar, Barcelona, Spain; j Hospital Universitario La Paz, Madrid, Spain; k Instituto de Investigacion Sanitaria del Hospital Universitario La Paz (IdiPAZ), Madrid, Spain; l Hospital Universitario Vall d'Hebron, Barcelona, Spain; m Instituto de Investigacion Sanitaria-Fundacion Jimenez Diaz (IIS-FJD), Madrid, Spain; n Hospital Universitario Fundación Jiménez Díaz, Madrid, Spain; o Hospital Clinico Universitario de Santiago, Santiago de Compostela, Spain; p Hospital Clinic, Institut d'Investigacions Biomèdiques August Pi i Sunyer (IDIBAPS), Universitat de Barcelona, Barcelona, Spain; q Hospital Clinico Universitario, Valencia, Spain; r Hospital Universitario y Politecnico La Fe, Valencia, Spain; s Hospital Universitari de Bellvitge, L’Hospitalet, Barcelona, Spain; t Catalan Institute of Oncology, L’Hospitalet, Barcelona, Spain; u Hospital General Universitario Gregorio Marañón, Madrid, Spain; v Instituto de Investigación Sanitaria Hospital Universitario Puerta de Hierro, Madrid, Spain; w Hospital Universitario Puerta de Hierro, Madrid, Spain; x Hospital General Universitario Dr. Balmis – Instituto de Investigación Sanitaria y Biomédica de Alicante (ISABIAL), Alicante, Spain; y Hospital de la Santa Creu i Sant Pau, Barcelona, Spain

**Keywords:** *RET* fusions, Next-generation sequencing, FISH, RT-PCR, Lung carcinoma

## Abstract

**Introduction:**

*RET* inhibitors with impressive overall response rates are now available for patients with NSCLC, yet the identification of *RET* fusions remains a difficult challenge. Most guidelines encourage the upfront use of next-generation sequencing (NGS), or alternatively, fluorescence in situ hybridization (FISH) or reverse transcriptase-polymerase chain reaction (RT-PCR) when NGS is not possible or available. Taken together, the suboptimal performance of single-analyte assays to detect *RET* fusions, although consistent with the notion of encouraging universal NGS, is currently widening some of the clinical practice gaps in the implementation of predictive biomarkers in patients with advanced NSCLC.

**Methods:**

This situation prompted us to evaluate several *RET* assays in a large multicenter cohort of *RET* fusion–positive NSCLC (n = 38) to obtain real-world data. In addition to RNA-based NGS (the criterion standard method), all positive specimens underwent break-apart *RET* FISH with two different assays and were also tested by an RT-PCR assay.

**Results:**

The most common *RET* partners were *KIF5B* (78.9%), followed by *CCDC6* (15.8%). The two *RET* NGS-positive but FISH-negative samples contained a *KIF5B(15)-RET(12)* fusion. The three *RET* fusions not identified with RT-PCR were *AKAP13(35)-RET(12)*, *KIF5B(24)-RET(9)* and *KIF5B(24)-RET(11)*. All three false-negative RT-PCR cases were FISH-positive, exhibited a typical break-apart pattern, and contained a very high number of positive tumor cells with both FISH assays. Signet ring cells, psammoma bodies, and pleomorphic features were frequently observed (in 34.2%, 39.5%, and 39.5% of tumors, respectively).

**Conclusions:**

In-depth knowledge of the advantages and disadvantages of the different *RET* testing methodologies could help clinical and molecular tumor boards implement and maintain sensible algorithms for the rapid and effective detection of *RET* fusions in patients with NSCLC. The likelihood of *RET* false-negative results with both FISH and RT-PCR reinforces the need for upfront NGS in patients with NSCLC.

## Introduction

The *RET* protooncogene is located on the long arm of chromosome 10 and encodes a transmembrane protein that consists of an extracellular ligand-binding domain, a transmembrane region, and an intracellular tyrosine kinase domain.^1^, ^2^, ^3^ RET activation occurs when the GDNF ligands bind to their receptors, causing homodimerization, autophosphorylation, and ultimately activation of downstream signaling pathways.^4^ Oncogenic activating fusions have been identified in a variety of malignant tumors, including papillary thyroid carcinomas and NSCLC.^4^^,^^5^ *RET* fusions are found in 1% to 2% of NSCLC, and there is a higher prevalence in never or light smokers, younger age, and adenocarcinoma (AC).^4^^,^^5^ In treatment-naive patients, *RET* fusions tend to be mutually exclusive with other major oncogenic drivers.^4^ The rearrangements typically involve the 3’ kinase domain of *RET* encoded by exons 12 to 18 to various 5’ heterologous upstream partner genes.^4^ In NSCLC, the most typically reported *RET* partners are *KIF5B* (∼70%), *CCDC6* (∼20%), and *NCOA4* (∼2%), and many other partners have been reported as isolated examples.^6^ Therefore, the molecular epidemiology of *RET* fusions is difficult to infer but the frequency of those uncommon *RET* partners with more overlap between the different series is usually around 1%: *ERC1, TRIM24, TRIM27, TRIM33, DOCK1, KIF13A,* and *KIAA1468*.^7^, ^8^, ^9^, ^10^, ^11^, ^12^, ^13^, ^14^, ^15^, ^16^ The development and approval of selective *RET* inhibitors in lung cancer, thyroid cancer or even in a tumor-agnostic strategy, with high efficacy, means that the relevance of accurately identifying *RET* fusions has never been greater.^4^^,^^5^^,^^12^^,^^15^, ^16^, ^17^, ^18^, ^19^, ^20^, ^21^, ^22^, ^23^, ^24^

The available diagnostic methodologies used to identify *RET* fusions include the increasingly popular next-generation sequencing (NGS) and single-gene approaches such as fluorescence in situ hybridization (FISH) and reverse transcriptase-polymerase chain reaction (RT-PCR).^20^^,^^25^ Accordingly, in clinical trials, there is vast heterogeneity in local testing methods, and between 18% to 42% of patients have been identified by either FISH or RT-PCR.^12^^,^^15^^,^^16^^,^^19^ Several professional organizations and academic groups have released recommendations on the standard methods to detect *RET* fusions in daily practice and clinical research.^6^^,^^10^^,^^26^^,^^27^ Most guidelines encourage the upfront use of NGS, or alternatively, FISH or RT-PCR when NGS is not possible or available.^6^^,^^10^^,^^26^ Although break-apart FISH has traditionally been the accepted standard test for the detection of fusions, *RET* FISH is especially difficult to interpret and may be susceptible to both false negatives and false positives.^10^ Moreover, the real-world performance of specific RT-PCR assays remains largely unknown.

Taken together, the suboptimal performance of single-analyte assays to detect *RET* fusions, although consistent with the notion of encouraging universal NGS, is currently widening some of the clinical practice gaps in the implementation of predictive biomarkers in advanced NSCLC.^28^ Therefore, we hypothesized that in-depth knowledge of the advantages and disadvantages of the different *RET* testing methodologies could help clinical and molecular tumor boards implement and maintain sensible algorithms for rapid and effective detection of predictive biomarkers (i.e., including *RET*) in patients with NSCLC. This situation prompted us to evaluate several *RET* assays (i.e., RNA-based NGS as criterion standard method, FISH, and RT-PCR) in a large multicenter cohort of *RET*-positive NSCLC to obtain real-world data.

## Materials and Methods

### Study Design and Tumor Samples

**Figure 1:**
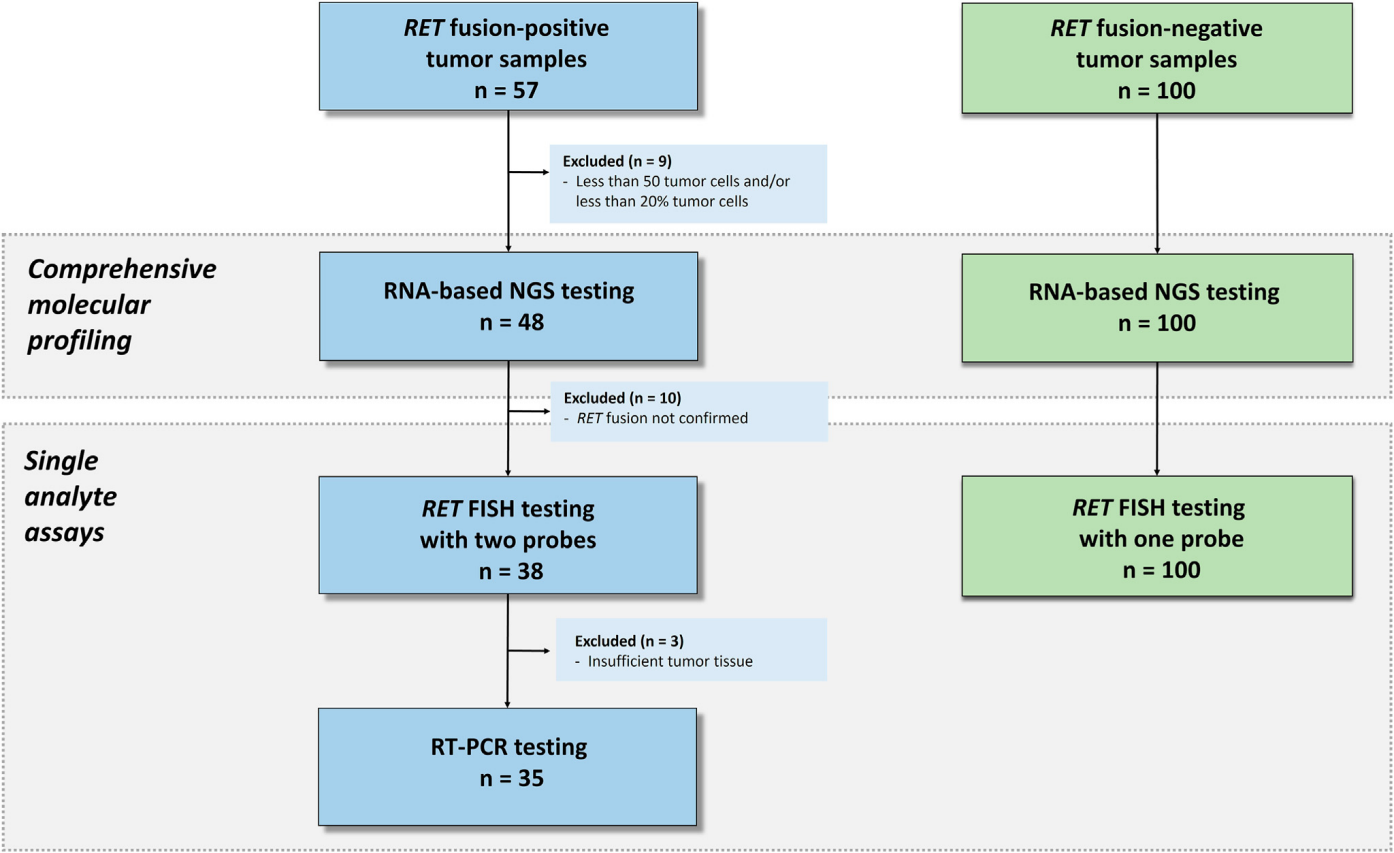
Flowchart of samples in the RETING study. FISH, fluorescence in situ hybridization; NGS, next-generation sequencing; RT-PCR, reverse transcriptase–polymerase chain reaction.

The flow diagram is depicted in Figure 1. There were 57 *RET* fusion–positive samples from patients with NSCLC that had been initially tested as part of routine clinical care in 16 different institutions, were used for this study (also known as RETING or RET and Individual gene assays & Next-Generation sequencing). To confirm the *RET* fusion–positive status, targeted RNA-based NGS analysis (the criterion standard method) was performed at the referral institution. Only cases with enough tissue available (i.e., a minimum of 20% tumor cell content) were included. In addition, 100 consecutive *RET* NGS-negative samples from NSCLC tested at the referral institution as part of routine clinical care were included as negative controls. The material available for all tumors was formalin-fixed and paraffin-embedded (FFPE). The specifics of formalin-fixation were unknown. All cases were reviewed by three pathologists (E.C., F.L.R., and J.L.R.C.). In addition to NGS, all positive specimens underwent break-apart *RET* FISH with two different assays using an automated scanning system and were also tested by an RNA PCR-based assay. In the negative cohort, only one *RET* FISH assay was investigated. The Institutional Ethics Committee at Fundacion de Investigation HM Hospitales and Hospital Universitario 12 de Octubre reviewed and approved this study. Each referring institution regulated the need for additional specific consent. Clinical data from the *RET* NGS-positive cohort were retrieved from the patient clinical records.

### NGS for RET Fusions

A targeted RNA-based NGS panel (Oncomine Comprehensive Assay v3 test [ThermoFisher Scientific, Waltham, MA]) was performed for all cases (positive and negative) on the Ion S5 sequencer with automated library preparation using the Ion Chef System, as described previously.^29^ For each FFPE tumor sample, freshly cut 5-μm–thick sections were collected on separate Eppendorf tubes for DNA and RNA extraction: three sections for surgical specimens and five sections for small biopsy specimens for each tube. The first and last sections were stained with hematoxylin-eosin and reviewed by two pathologists (E.C. and F.L-R.) to confirm that the percentage of tumor cells was greater than or equal to 20%. The DNA extraction was performed with the Cobas DNA Sample Preparation Kit (Roche Molecular Systems, Pleasanton, CA) following the manufactureŕs instructions. The RNA extraction was performed with the High Pure FFPET RNA Isolation Kit (Roche Molecular Systems) following the manufacturer’s instructions. The RNA was then purified and concentrated by using the GeneJET RNA cleanup and concentration micro kit (ThermoFisher Scientific). The protocol for the NGS analyses followed the manufactureŕs instructions, and a minimum of 500,000 mapped fusion panel reads was required for *RET* fusion analysis. The *RET* NGS result was used as the criterion standard method and the complete NGS report was only available for the *RET* NGS-positive cohort.

### FISH for RET fusions

FISH was carried out on unstained 4-μm–thick FFPE tumor tissue sections from all cases. For all positive cases, we used two commercial break-apart *RET* FISH assays: Vysis *RET* FISH Break-Apart Probe RUO kit (Abbott Molecular, IL) and ZytoLight SPEC *RET* Dual Color BreakApart Probe (ZytoVision GMbH, Bremerhaven, Germany). In the negative cohort, we only investigated the Vysis *RET* FISH probe. The methodologies have been described in detail elsewhere.^30^^,^^31^ *RET* FISH assays were independently captured and scored with the automated BioView Duet scanning system (BioView, Rehovot, Israel) by a thoracic pathologist (E.C.) or molecular biologist (S.H.). A minimum of 50 tumor nuclei were counted. *RET* FISH-positive cases were defined as those with greater than or equal to 15% break-apart signals (separated by more than one signal diameter) or isolated 3’ signals in tumor cells.^26^^,^^32^^,^^33^ Using our own prespecified criteria, if the separation between the signals was greater than one but less than two signal diameters, the pattern was named “borderline positive break-apart.” *RET* FISH-negative samples were defined as those with fusion signals, isolated 5’ signals, or less than 15% of positive cells.^26^^,^^32^^,^^33^

### RT- PCR Assay for RET Fusions

The AmoyDx Multigene Mutations Detection Kit (Amoy Diagnostics, Xiamen, People's Republic of China) was performed for all positive samples, according to the manufacturer's instructions. This RNA-based assay is designed to detect six different *RET* fusion variants (i.e., *CCDC6[1]-RET[12], NCOA4[6]-RET[12], KIF5B[15]-RET[12], KIF5B[16]-RET[12], KIF5B[22]-RET[12] and KIF5B[23]-RET[12]*) on a Cobas z 480 (user-defined function channel) instrument.

## Results

The clinicopathologic characteristics of patients with *RET* fusions are presented in Table 1.

**Table 1:** Clinicopathologic Features of Patients with *RET* Fusions

Characteristic	Patients, n (%)^a^ N = 38
Tumor histology	
AC	35 (92.1)
NSCLC-NOS	3 (7.9)
Specimen type	
Surgical	20 (52.6)
Small biopsy	15 (39.5)
Cell block	3 (7.9)
Age at diagnosis, yr^a^	
Median (range)	65 (39-89)
Distribution	
≥18 to 64 yr	17 (45.9)
≥65 yr	20 (54.1)
Sex^a^	
Female	26 (70.3)
Male	11 (29.7)
Smoking history^a^	
Never smoked	26 (70.3)
Current / former smoker	11 (29.7)
Stage at initial diagnosis^a^	
I	6 (16.2)
II	4 (10.8)
III	6 (16.2)
IV	21 (56.8)
Metastasis sites for stage IV disease^a^	
Multiple organs	16 (57.1)
Lung	15 (53.6)
Bone	13 (46.4)
Lymph node	9 (32.1)
Liver	7 (25)
Brain	6 (21.4)
Pleura	5 (17.9)
Others	4 (14.3)
No. of previous lines before *RET* TKI therapy^a^^,^^b^	
0	7 (25)
1	15 (53.6)
2	3 (10.7)
≥3	3 (10.7)
*RET* TKI therapy^a^^,^^b^	
Pralsetinib	10 (35.7)
Selpercatinib	6 (21.4)
Others	5 (17.9)
None	7 (25)
Best overall response after *RET* TKI therapy^a^^,^^c^	
Complete response	3 (14.3)
Partial response	10 (47.7)
Stable disease	2 (9.5)
Progressive disease	4 (19)
Not available	2 (9.5)

AC, adenocarcinoma; NSCLC-NOS, non-small cell lung carcinoma, not otherwise specified; TKI, tyrosine kinase inhibitor.

a Clinical information was available for 37 patients.

b Patients with stage IV disease (n=28).

c Patients with stage IV disease treated with *RET* TKI therapy (n=21).

### RET Fusions Assessed by NGS

Of the 57 *RET* fusion–positive lung carcinoma specimens, nine cases (9 of 57, 15.8%) were excluded for lack of sufficient tumor content (see above). Six samples (6 of 48, 12.5%) were negative for *RET* fusions and results could not be assessed in four cases (4 of 48, 8.3%) owing to insufficient sequencing coverage (Fig. 1). Therefore, the final size of the positive cohort was 38 tumors. There were 30 cases (30 of 38, 78.9%) that had a *KIF5B-RET* fusion (25 cases corresponding to the *KIF5B[15]-RET[12]* variant, two corresponding to the *KIF5B[16]-RET[12]* variant, and the remaining three cases corresponding to *KIF5B[23]-RET[12]*, *KIF5B[24]-RET[11]* and *KIF5B*[24]-RET*[9]* variants, respectively), six cases (6 of 38, 15.8%) exhibited a *CCDC6(1)-RET(12)* fusion, one tumor (1 of 38, 2.6%) presented a *NCOA4(6)-RET(12)*, and one sample (1 of 38, 2.6%) contained a *AKAP13(35)-RET(12)* fusion. Non-*RET* alterations were present in 44.7% (17 of 38) of *RET-*positive patients. The three more common co-occurring gene variants included *TP53* (5 of 38, 13.2%), *SETD2* (5 of 38, 13.2%), and *CTNNB1* (2 of 38, 5.3%) mutations. Interestingly, isolated examples of copy number variations in genes *MDM2* (1 of 38, 2.6%) and *CDK6* (1 of 38, 2.6%) were also identified.

Because of the retrospective nature of the negative cohort, NGS had been successful in all 100 *RET*-negative tumors (Fig. 1).

### RET Fusions Assessed by FISH

**Figure 2:**
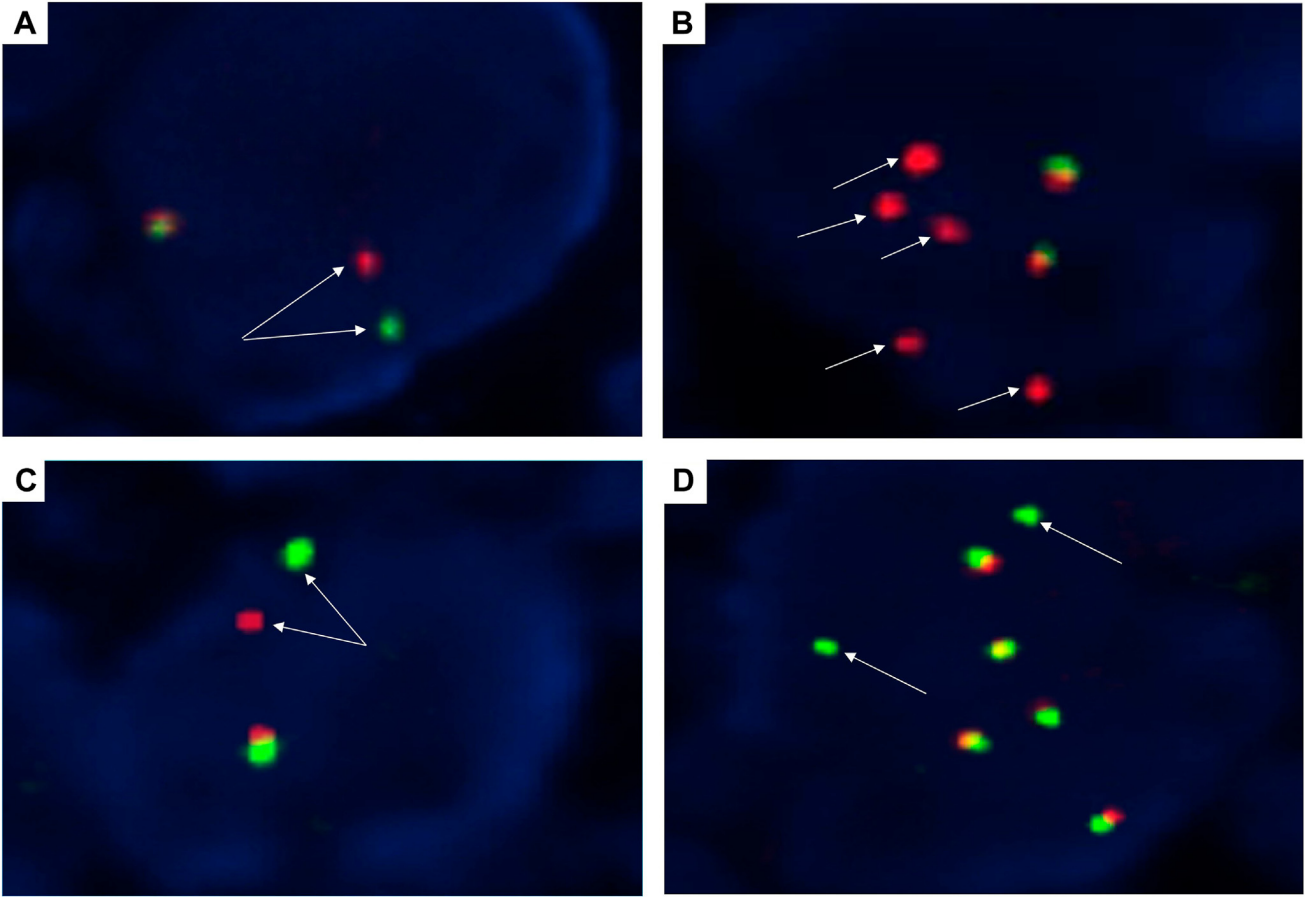
Representative examples of *RET* FISH-positive NSCLCs using the Vysis *RET* Probe (*A,B*) and the ZytoVision *RET* Probe (*C,D*). (*A,C*) A typical break-apart pattern is shown with one fused signal and one break-apart signal per nucleus (arrows). (*B,D*) An isolated 3’ signal pattern is depicted (red signals with the Vysis probe and green signals with the ZytoVision probe) (arrows). All four cases were scored using the BioView Duet scoring system and were *RET* NGS-positive. See text for details. Original magnification: x1000. FISH, fluorescence in situ hybridization; NGS, next-generation sequencing.

**Figure 3:**
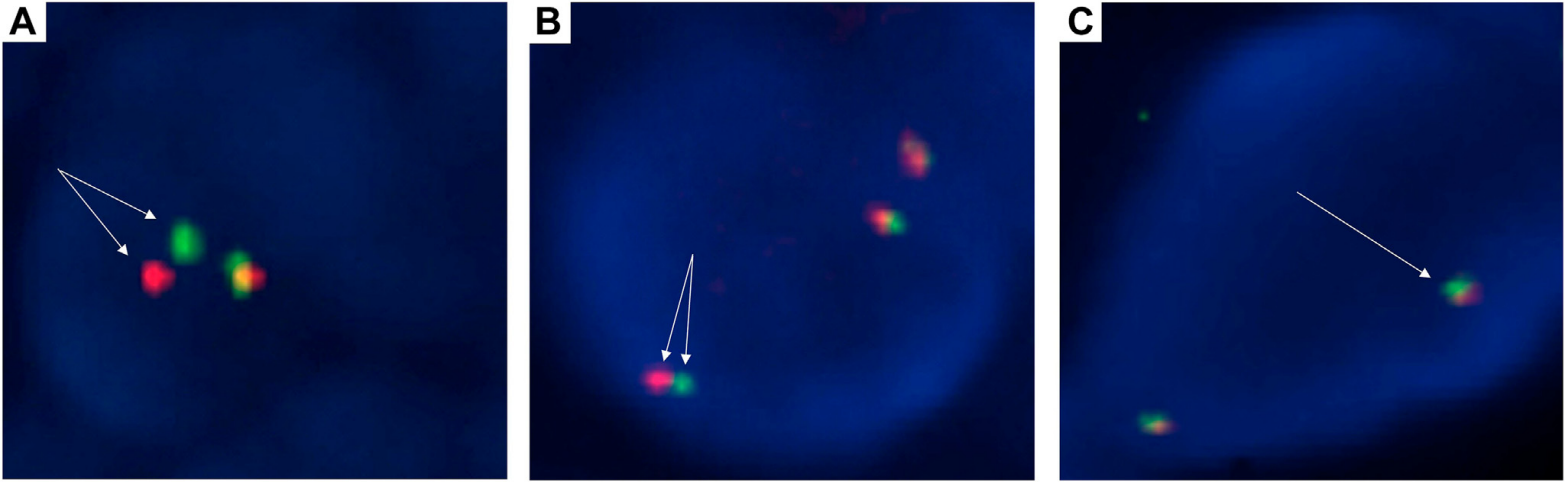
Representative examples of *RET* FISH patterns: borderline break-apart positive (*A*), false-negative (*B*), and (*C*) typical negative fusion signal pattern (*C*). (*A*) A tumor with a *CCDC6-RET* fusion showing a borderline break-apart positive pattern. (*B*) A tumor with a *KIF5B-RET* fusion showing insufficient separation between the red and the green signals (i.e., FISH false-negative). (*C*) A typical example of a tumor without *RET* fusions exhibits two fused signals. All images correspond to the Vysis *RET* probe and were interpreted using the BioView Duet scoring system. The fusion status was confirmed by NGS. See text for details. Original magnification: x1000. FISH, fluorescence in situ hybridization.

All 138 specimens (positive and negative) were successfully tested by FISH (Fig. 1). In agreement with the NGS results, 36 out of the 38 (94.7%) *RET* NGS-positive samples were *RET* FISH-positive by both probes. The overall results were very similar for both probes. The mean percentage of positive cells was 74.6% (median 77%, range 16%–100%) using the Vysis probe and 70.5% (median 74%, range 18%-96%) with the ZytoVision probe. The break-apart pattern was more frequently observed than the isolated 3’ signal pattern (30 of 36, 83.3% versus 6 of 36, 16.7%) (Fig. 2*A–D*). The number of cases with a borderline positive break-apart pattern (see definition above) was higher with one of the probes (13 of 36, 36.1% for ZytoVision versus 6 of 36, 16.7% for Vysis). Interestingly, this borderline pattern was identified in all fusion partners except *AKAP13.* The frequencies were higher for *CCDC6* (3 of 6, 50% with ZytoVision and 1 of 6, 16.7% with Vysis) than for *KIF5B* (9 of 28, 32.1% with ZytoVision and 4 of 28, 14.3% with Vysis) (Fig. 3*A*). The two *RET* NGS-positive but FISH-negative samples contained a *KIF5B(15)-RET(12)* fusion (Fig. 3*B*). Both ACs exhibited psammoma bodies and were diagnosed in a surgical specimen. These two patients received a *RET* TKI and had partial responses. The FISH results for all cases from the negative cohort agreed with those obtained by NGS (Fig. 3*C*).

### RET Fusions Assessed by RT-PCR

Three *RET* NGS-positive cases were excluded for lack of tumor tissue after the previous analyses (Fig. 1). There were 32 out of the remaining 35 (32 of 35, 91.4%) NGS-positive samples that were RT-PCR–positive. The three *RET* fusions not identified with RT-PCR were *AKAP13(35)-RET(12)*, *KIF5B(24)-RET(9)* and *KIF5B(24)-RET(11)*. All three cases were AC that were diagnosed by surgical specimens (n = 2) or core-needle biopsy (n = 1). Both surgical specimens contained either signet ring cells or psammoma bodies. All three cases were FISH-positive, exhibited a typical break-apart pattern, and contained a very high number of positive tumor cells with both FISH assays (82%, 92%, and 90% for Vysis; 60%, 94%, and 96% for ZytoVision, respectively). Of note, two of these three patients received a *RET* TKI and had partial responses.

### Histologic Characteristics

**Figure 4:**
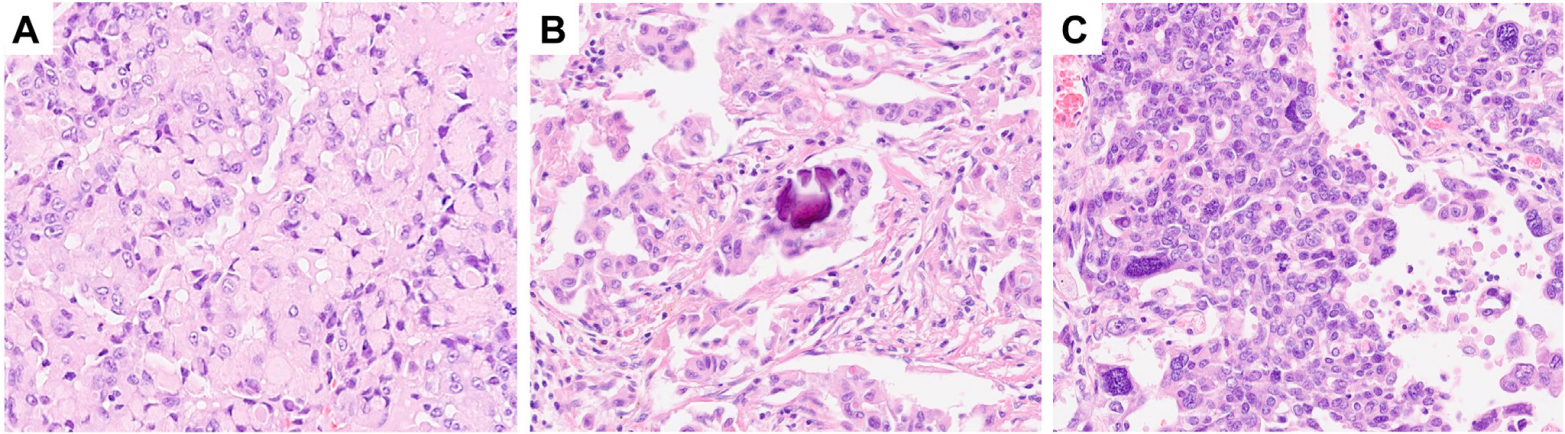
Typical features of NSCLC with *RET* fusions. (*A*) signet ring cells, (*B*) psammoma bodies, and (*C*) pleomorphic nuclei (hematoxylin-eosin, original magnification X200 [*A-C*]).

A total of 35 tumors (35 of 38, 92.1%) were AC and three (3 of 38, 7.9%) were NSCLC not otherwise specified. Of the AC, 16 (45.7%) were observed to have a predominant acinar pattern, 11 (31.4%) presented a solid architecture, five (14.3%) had a predominant lepidic pattern, two (5.7%) exhibited a papillary growth (*KIF5B[15]-RET[12]* and *CCDC6[1]-RET[12]*), and one (2.8%) had a predominant micropapillary pattern (*KIF5B[15]-RET[12]*). Signet ring cells, psammoma bodies, and pleomorphic features were frequently observed (in 13 of 38 [34.2%], 15 of 38 [39.5%], and 15 of 38 [39.5%] of tumors, respectively) (Fig. 4*A–C*). Interestingly, pleomorphism was only present with the *KIF5B* partner.

## Discussion

The information presented herein is very timely because a recent survey from more than 500,000 patients has identified that almost 50% of patients with advanced NSCLC were not candidates for targeted therapies because of biomarker testing issues.^28^ The clinical gaps can be summarized as follows: tissue (insufficient tissue or inaccurate estimation of tumor cell content), testing (appropriate assay was not ordered or results were inconclusive or false-negative), and time (turnaround time delays).^28^ Therefore, in some series the frequency of *RET* fusions falls below 1%^11^^,^^34^, ^35^, ^36^ and, unsurprisingly the percentage is within the expected range (i.e., 1%-2%) in fully genotyped cohorts.^36^ These results are consistent with mounting evidence of similar trends for other actionable fusions.^37^, ^38^, ^39^ Although broad molecular profiling is the recommended NSCLC testing option in most guidelines, NGS is not universally available or requested.^6^^,^^26^^,^^39^, ^40^, ^41^, ^42^ Until NGS is routinely performed in all patients with advanced NSCLC, a deep understanding of the concept of “molecular redundancy” is reassuring.^43^ This notion has been recommended and endorsed by all the major professional organizations in the field and can be summarized as follows: “Laboratories should ensure that test results that are unexpected, discordant, equivocal, or otherwise of low confidence are confirmed or resolved using an alternative method or sample.”^43^ Therefore, in this RETING study, we wanted to explore the performance of typically used single-gene *RET* assays as potential complementary tools to NGS in testing workflows for patients with advanced NSCLC.^6^^,^^10^^,^^26^

Reasoning that RNA sequencing is now becoming the accepted standard for fusion identification, because of its superior sensitivity,^40^^,^^44^ we decided to use as our standard criterion a large RNA-based NGS assay that required very little input RNA. The molecular landscape of *RET* fusions in our series is remarkably similar to previous reports (i.e., high frequency of co-occurring *TP53*, *SETD2,* and *CTNNB1* mutations*)*,^7^^,^^8^^,^^10^^,^^45^, ^46^, ^47^, ^48^ including the puzzling finding of *MDM2* and *CDK4/6* amplifications.^8^^,^^46^ Overall, the variety and individual frequencies of *RET* partners identified were like those described (Table 2).^7^, ^8^, ^9^, ^10^, ^11^^,^^13^^,^^14^^,^^45^, ^46^, ^47^^,^^49^, ^50^, ^51^, ^52^, ^53^, ^54^ The most common fusion partners are *KIF5B*, *CCDC6* and *NCOA4.* Several conclusions can be drawn from our study. First, the performance of two typically used FISH probes was similar: two clear-cut false-negative results with both probes on the same samples. It is unfortunate that both suboptimal readings involved the most frequent *RET* fusion in patients with NSCLC (i.e., *KIF5B[15]-RET[12]* fusion) (Table 2). Despite the initial description that *RET* FISH false-negative results were restricted to the *NCOA4* partner,^10^ isolated examples involving *KIF5B* fusions have been reported.^33^^,^^46^^,^^47^^,^^55^ Second, our absence of *RET* FISH false-positive results could be because of the use of an outstanding automated FISH scanning system and a large NGS panel as a FISH comparator. In agreement with other authors, we believe that the current false-positive rate of *RET* FISH could be overestimated for two main reasons: (1) the adoption of a low threshold of signal separation for positive break-apart signals or a low percentage of positive nuclei as the cutoff for positivity,^56^ and (2) the use of RT-PCR or small NGS panels as a standard criterion, which may miss some fusion partners.^33^^,^^53^^,^^57^ Moreover, similarly to other break-apart FISH probes,^58^ the presence of complex patterns in *RET* FISH assays (e.g., loss of signals) is clearly linked to false-positive results.^10^^,^^32^^,^^33^ Nevertheless, the literature on this topic should be interpreted with great caution because most series are small, and very different methods and criteria have been used (Table 3).^10^^,^^32^^,^^33^^,^^46^, ^47^, ^48^^,^^53^^,^^59^, ^60^, ^61^, ^62^, ^63^

**Table 2:** Summary of Studies Addressing the Tissue Detection Rate of *RET* Fusions in Patients with NSCLC^a^

Study	No. of Patients with Identified Upstream Partners	Frequencies of *RET* Partners Genes (%)			Representation of *RET* Fusions not Identified by Single-Gene Assays in the Current Study (%^b^)			
					FISH False-Negative	RT-PCR False-Negative		
		*KIF5B*	*CCDC6*	*NCOA4*	*KIF5B(15)-RET(12 )*	*KIF5B(24)-RET(9)*	*KIF5B(24)-RET(11)*	*AKAP13(35)-RET(12)*
Parimi et al.^8^ 2023	523	66	18.2	2.9	N/A	N/A	N/A	N/A
Wang *et al.*^49^ 2022	262	48.5	16	2.3	N/A	N/A	N/A	0
Feng *et al.*^47^ 2022	167	68.2	16.8	1.2	N/A	N/A	N/A	0
Aldea *et al.*^7^ 2023	166	72	17	1.6	N/A	N/A	N/A	0
Yang *et al.*^10^ 2021	99	68.7	14	3	55.6	0	1	0
Gautschi *et al.*^50^ 2017	81	72	23	2	N/A	N/A	N/A	0
Illini *et al.*^14^ 2021	50	66	20	2	N/A	N/A	N/A	N/A
Meng *et al.*^51^ 2022	49	26.5	12.2	2.1	N/A	N/A	N/A	0
Hess *et al.*^11^ 2021	46	63	23.9	6.5	N/A	N/A	N/A	0
Xiang *et al.*^9^ 2022	41	68	12	0	56.1	0	0	2,4^c^
Tan *et al.*^46^ 2020	40	62.5	30	0	N/A	N/A	N/A	0
Passaro *et al.*^54^ 2022	34	55.7	9.8	3.3	N/A	N/A	N/A	N/A
Gao *et al.*^45^ 2023	29	62	21	0	55.2	0	0	0
Qiu *et al*.^52^ 2020	23	60.9	26.1	4.3	26.1	0	0	0
Jeon *et al.*^13^ 2023	23	69.6	21.7	4.3	N/A	N/A	N/A	N/A
Tsuta *et al.*^53^ 2014	22	86.4	13.6	0	62,5^d^	0^d^	0^d^	0^d^
Conde *et al.* 2024	38	78.9	15.8	2.6	65.8	2.6	2.6	2.6

NSCLC, non-small cell lung carcinoma.

a Only studies with more than 20 *RET*-positive cases are included.

b The denominator is the total number of *RET* fusions.

c Corresponds to a *AKAP13(35)-RET(11)*.

d The specific breakpoint is only available for 16 of the 22 *RET* fusions.

**Table 3:** Summary of Studies Addressing the use of FISH to Detect *RET* Fusions in Patients with NSCLC^a^

Study	No. of Patients with *RET* Fusions	Genomic Confirmation	Probe	No. of Cells Evaluated	Cut-off for Positivity (%)	Range of Positive Signals (%)	Types of Positive Signals			Inclusion of an Equivocal Category
							BA Signals	Distance Between BA Signals (Signal Diameter)	Isolated Signals	
Yang *et al.*^10^ 2021	48	Yes	ZytoVision	100	≥10	N/A	Yes	≥2	Yes	No
Feng *et al.*^47^ 2022	25	Yes	Other	>100	≥15	N/A	Yes	N/A	Yes	No
Baker *et al.*^33^ 2022	23	Yes	Vysis	50	≥19	13-73	Yes	>1	Yes	Yes
Michels *et al.*^61^ 2016	22	No^b^	ZytoVision	100	≥20 and ≥15	21-100	Yes	N/A	Yes	No
Tsuta *et al.*^53^ 2014	22	Yes	Other	50	≥20	22-72	Yes	>1	Yes	No
Radonic *et al.*^32^ 2021	18	Yes	Several	50	≥15	N/A	Yes	>1	Yes	Yes
Conde *et al.* 2024	38	Yes	Vysis / ZytoVision	50	≥15	16-100 / 18-96	Yes	>1	Yes	Yes

BA, break-apart; NSCLC, non-small cell lung carcinoma.

a Only series with genomic confirmation of more than 15 cases and reproducible FISH protocols are included.

b Despite of the lack of genomic confirmation this series is included due to the high quality of the FISH data.

When using RT-PCR it is important to understand the concept of “diagnostic sensitivity,” which relates to the comprehensiveness of the assay, or the percentage of all *RET* fusions described for the gene detectable by the given assay.^6^^,^^64^ Users of these assays should be constantly aware that “pseudo false-negatives” (i.e., because fusion partners are not included in the design of an assay) are unavoidable. Accordingly, three *RET* fusions were missed by the RT-PCR kit, emphasizing the need to always consider NGS testing in patients with driver-negative NSCLC.^6^ A review of the literature in light of our findings suggests that the presence of an *AKAP13* partner is a rare event. Unfortunately, the lack of detail regarding the specific *KIF5B* breakpoints in some large series prevents drawing definitive conclusions regarding the molecular epidemiology of *KIF5B(24)-RET(9)* and *KIF5B(24)-RET(11)* fusions (Table 2). According to Mizukami et al.,^65^ the frequency of the *KIF5B(24)-RET(11)* fusion across several cohorts comprising 60 patients is 2%, which is similar to our experience (2.6%). Nevertheless, the occasional presence of this fusion in two very small series (13 and 14 patients with a frequency of around 7%) remains worrisome and highlights the difficulty in calculating the risk of false-negative results when using RT-PCR for *RET* testing.^59^^,^^62^ Single-analyte assays are still very popular across the globe for cost reasons or because exclusionary testing is implemented in high *EGFR* mutation prevalence regions.^6^ In exclusionary testing, several biomarkers are tested first, followed by NGS in driver-negative patients. Despite contradictory reports on the cost-effectiveness of this strategy,^66^, ^67^, ^68^ recently released expert consensus or recommendations from the Asia-Pacific region support the use of upfront NGS in patients with NSCLC.^27^^,^^69^

Although RET immunohistochemistry to detect *RET* fusions is not currently recommended because of its wide range of sensitivity (50%–100%) and specificity (30%–90%),^6^^,^^10^^,^^26^^,^^27^ several comments might be helpful for the future implementation and development of RET antibodies: (1) evidence on the topic is still inconclusive because of the small sample size of many reports and the insufficient representation of non-*KIF5B* partners^26^; (2) only antibodies directed to the C-terminal portion of RET should be used to identify the chimeric protein^26^; and (3) the clone EPR2871 is probably the most frequently used and well characterized, with an interesting association between the fusion partner and the expression of the protein.^10^ Some authors have reported higher H-scores for *KIF5B* fusions, which resulted in perfect sensitivity for the detection of *KIF5B-RET* fusions.^10^

The histologic characteristics of our NSCLC with *RET* fusions is concordant with the literature. A careful review of published studies identifies that most cases are AC (range: 82%–100%, mean: 92.6%, median: 94%).^7^, ^8^, ^9^, ^10^^,^^45^^,^^47^^,^^50^, ^51^, ^52^, ^53^^,^^62^^,^^63^ That *RET* fusion–positive AC can contain signet ring cells (range: 27-36%, mean: 30.7%, median: 30%) and psammoma bodies are well known,^62^^,^^70^ but the predictive value of these features is not fully recognized in clinical practice. Of note, four of the five false-negative FISH/RT-PCR samples contained either signet ring cells or psammoma bodies. Accordingly, pathologists should always report them and persevere in the search for actionable fusions in those circumstances, as they can also be found in NSCLC with *ALK* or *ROS1* fusions.^30^^,^^31^^,^^70^ Another interesting and underrecognized feature is the presence of papillary or micropapillary patterns in *RET* fusion–positive lung AC: almost 9% of the AC in the present series exhibited either one and reported rates to range from 9% to 36% (median: 20%, media: 22%).^48^^,^^62^^,^^63^ In agreement with other authors, both *KIF5B* and non-*KIF5B* partners were involved in papillary formation.^62^^,^^63^^,^^71^ Finally, it is important to emphasize that *RET* fusions have been reported in other lung carcinoma subtypes, including squamous cell carcinomas,^8^^,^^18^^,^^50^^,^^52^^,^^72^^,^^73^ adenosquamous carcinomas,^8^^,^^47^^,^^52^^,^^63^^,^^73^ sarcomatoid carcinomas,^51^ pleomorphic carcinomas,^10^ and neuroendocrine carcinomas.^7^, ^8^, ^9^, ^10^^,^^18^^,^^52^^,^^72^^,^^74^ Interestingly, neuroendocrine differentiation can also be found in pancancer studies of *RET* fusion–positive solid tumors, highlighting the need to also use histologic classification as a way to increase the likelihood of finding an actionable fusion in tumor-agnostic approaches, as counterintuitive as it might seem.^21^^,^^22^^,^^29^^,^^49^

In conclusion, the potential for false-negative results with single-analyte assays reinforces the need for upfront NGS in patients with NSCLC. A consideration of the clinical problem of NSCLC highlights the need to be aware of how the methods that we use perform in the real-world setting.

## CRediT Authorship Contribution Statement

**Esther Conde:** Conceptualization; Data curation; Formal analysis; Funding acquisition; Investigation; Methodology; Resources; Supervision; Visualization; Roles/Writing - original draft; Writing - review & editing.

**Susana Hernandez:** Conceptualization; Data curation; Formal analysis; Funding acquisition; Investigation; Methodology; Resources; Supervision; Visualization; Roles/Writing - original draft; Writing - review & editing.

**Jose Luis Rodriguez Carrillo:** Formal analysis; Investigation; Methodology; Visualization; Review & editing.

**Rebeca Martinez:** Review & editing.

**Marta Alonso**: Review & editing.

**Daniel Curto:** Clinical data compilation, review & editing.

**Beatriz Jimenez**: Resources; Review & editing.

**Alejandra Caminoa**: Resources; Review & editing.

**Amparo Benito**: Resources; Review & editing.

**Pilar Garrido**: Resources; Review & editing.

**Sergi Clave**: Resources; Review & editing.

**Edurne Arriola**: Resources; Review & editing.

**Isabel Esteban-Rodriguez**: Resources; Review & editing.

**Javier De Castro**: Resources; Review & editing.

**Irene Sansano**: Resources; Review & editing.

**Enriqueta Felip**: Resources; Review & editing.

**Federico Rojo**: Resources; Review & editing.

**Manuel Dómine**: Resources; Review & editing.

**Ihab Abdulkader**: Resources; Review & editing.

**Jorge Garcia-Gonzalez**: Resources; Review & editing.

**Cristina Teixido**: Resources; Review & editing.

**Noemi Reguart**: Resources; Review & editing.

**Desamparados Compañ**: Resources; Review & editing.

**Amelia Insa**: Resources; Review & editing.

**Nuria Mancheño**: Resources; Review & editing.

**Sarai Palanca**: Resources; Review & editing.

**Oscar Juan-Vidal**: Resources; Review & editing.

**Nuria Baixeras:** Resources; Review & editing.

**Ernest Nadal**: Resources; Review & editing.

**Maria Cebollero**: Resources; Review & editing.

**Antonio Calles**: Resources; Review & editing.

**Paloma Martin**: Resources; Review & editing.

**Clara Salas**: Resources; Review & editing.

**Mariano Provencio**: Resources; Review & editing.

**Ignacio Aranda**: Resources; Writing - review & editing.

**Bartomeu Massuti**: Resources; Review & editing.

**Laura Lopez-Vilaro:** Resources; Review & editing.

**Margarita Majem**: Resources; Writing - review & editing.

**Luis Paz-Ares**: Resources; Review & editing.

**Fernando Lopez-Rios:** Conceptualization; Data curation; Formal analysis; Funding acquisition; Investigation; Methodology; Resources; Supervision; Visualization; Roles/Writing - original draft; Writing - review & editing.

## Disclosure

Dr. Conde has received research funding from Eli Lilly, 10.13039/100004325AstraZeneca, and 10.13039/100011033ThermoFisher Scientific; and honoraria from Pfizer, Roche, AstraZeneca, Janssen, and Eli Lilly. Dr. Hernandez has received research funding from Eli Lilly, AstraZeneca, and ThermoFisher Scientific, and honoraria from Pfizer, Roche, AstraZeneca, ThermoFisher Scientific, and Eli Lilly. Mr. Alonso has received research funding from AstraZeneca, and honoraria from Pfizer, Roche, and AstraZeneca. Dr. Jimenez has received honoraria from Roche. Dr. Garrido has received research grants from 10.13039/100002429Amgen, AstraZeneca, Blueprint, 10.13039/100002491Bristol-Myers Squibb, Boehringer Ingelheim, 10.13039/501100002973Daiichi-Sankyo, 10.13039/100004330GlaxoSmithKline, 10.13039/100005565Janssen, IO Biotech, Eli Lilly, 10.13039/100009947Merck Sharp & Dohme, 10.13039/100004337Roche, Takeda; and honoraria from AbbVie, Amgen, AstraZeneca, Bayer, Bristol-Myers Squibb, Boehringer Ingelheim, Daiichi-Sankyo, GlaxoSmithKline, Janssen, Eli Lilly, Merck Sharp & Dohme, Novartis, Pfizer, Roche, Sanofi, Takeda, Medscape, and Touch Medical. Dr. Clave has received honoraria from AstraZeneca, Pfizer, Roche, Eli Lilly, and Takeda. Dr. Arriola has received honoraria from AstraZeneca, Boehringer Ingelheim, Pfizer, Roche/Genentech, Eli Lilly and Company, Novartis, Takeda, Merck Sharp & Dohme, Bayer, and Bristol Myers Squibb. Dr. Esteban-Rodriguez has received honoraria from AstraZeneca, Pfizer, and Merck Sharp & Dohme. Dr. De Castro has received honoraria from AstraZeneca, Bristol Myers Squibb, Hoffmann- La Roche, Merck Sharp and Dohme, Boehringer-Ingelheim, Janssen, Eli Lilly, Sanofi, Takeda, Pfizer, Glaxo, and Gilead. Dr. Sansano has received honoraria from F. Hoffmann La Roche AG, Merck Sharp & Dohme, Pfizer, Takeda, AstraZeneca, and Boehringer Ingelheim. Dr. Felip has received honoraria from AbbVie, Amgen, AstraZeneca, Bayer, Beigene, Boehringer Ingelheim, Bristol Myers Squibb, Daiichi-Sankyo, Eli Lilly, F. Hoffmann – La Roche, Gilead, Glaxo Smith Kline, Genentech, Janssen, Medical Trends, Medscape, Merck Serono, Merck Sharp & Dohme, Novartis, Peptomyc, Peervoice, Pfizer, Regeneron, Sanofi, Takeda, Turning Point, and Touch Oncology. Dr. Rojo has received research funding from 10.13039/100004337Roche, AstraZeneca, Menarini, 10.13039/100004336Novartis, 10.13039/100004334Merck, Merck Sharp & Dohme, Bristol-Myers Squibb, 10.13039/100004319Pfizer, GlaxoSmithKline, Palex, Amgen, 10.13039/100004322Agilent, and Janssen, and honoraria from Roche, AstraZeneca, Menarini, Novartis, Merck, Merck Sharp & Dohme, Bristol-Myers Squibb, Pfizer, GlaxoSmithKline, Palex, Amgen, Agilent, Janssen. Dr. Dómine has received honoraria from AstraZeneca, Boehringer Ingelheim, Pfizer, Roche/Genentech, Takeda, Merck Sharp & Dohme, and Bristol Myers Squibb. Dr. Abdulkader has received honoraria from AstraZeneca, Eli Lilly, Pfizer, Roche, Merck Sharp & Dohme, Bristol Myers Squibb, Takeda, and Agilent Technologies S.A. Dr. Garcia-Gonzalez has received honoraria from Amgen, AstraZeneca, Boehringer Ingelheim, Bristol Myers Squibb, Merck Sharp & Dohme, Novartis, Roche, Sanofi, Pierre Fabre, Eli Lilly, Pfizer, and Takeda. Dr. Teixido has received honoraria from Novartis, AstraZeneca, Roche, Merck Sharp Dohme, Pfizer, Janssen, Eli Lilly, and, Bristol Myers Squibb. Dr. Reguart has received honoraria from Amgen, AstraZeneca, Bayer, Bristol-Myers Squibb, Boehringer, Guardant, Janssen, Merck Sharp & Dohme, Novartis, Pfizer, Roche, Sanofi, and Takeda. Dr. Insa has received honoraria from Roche, Bristol Myers Squibb, Sanofi, Pfizer, Boehringer Ingelheim, AstraZeneca, Takeda, Bayer, Merck Sharp & Dohme, and Eli Lilly. Dr. Mancheño has received honoraria from Roche, AstraZeneca, and Pfizer. Dr. Palanca has received honoraria from Roche Pharma, Pfizer, Amgen, AstraZeneca, Takeda, Eli Lilly, and Janssen. Dr. Juan-Vidal has received honoraria from Boehringer Ingelheim, Bristol Myers Squibb, Merck Sharp & Dohme, Roche/Genetech, AstraZeneca, Pfizer, Eli Lilly, and Takeda. Dr. Baixeras has received honoraria from AstraZeneca and Eli Lilly. Dr. Nadal has received research funding from Roche, Pfizer, Bristol-Myers Squibb and Merck Serono, and honoraria from Roche, Bristol Myers Squibb, Merck Sharp Dohme, Merck Serono, Sanofi, Pfizer, Eli Lilly, Janssen, Amgen, Daiichi-Sankyo, Boehringer Ingelheim, AstraZeneca, Takeda, Sanofi, Pierre Fabre, Qiagen, Janssen, and Bayer. Dr. Calles has received research funding from Merck Sharp & Dome, and honoraria from AstraZeneca, Boehringer Ingelheim, Pfizer, Roche/Genentech, Eli Lilly and Company, Novartis, Takeda, Merck Sharp & Dohme, and Bristol Myers Squibb. Dr. Martin has received honoraria from Daiichi-Sankyo and Pfizer. Dr. Salas has received honoraria from Boehringer Ingelheim, Pfizer, and Merck Sharp & Dohme. Dr. Provencio has received honoraria from AstraZeneca, Boehringer Ingelheim, Pfizer, Roche/Genentech, Takeda, Merck Sharp & Dohme, and Bristol Myers Squibb. Dr. Massuti has received research funding from Bristol Myers Squibb, and honoraria from Bristol Myers Squibb, Roche, Janssen, Merck Sharp & Dohme, and AstraZeneca. Dr. Majem has received research funding from 10.13039/100002429Amgen Inc., AstraZeneca, Bristol Myers Squibb, and Roche, and honoraria from AstraZeneca, Bayer, Boehringer Ingelheim, Bristol Myers Squibb, Kyowa Kyrin, Merck Sharp & Dohme, Novartis, Pierre Fabre, Roche, Sanofi, and Takeda. Dr. Paz-Ares has received research funding from Merck Sharp & Dohme, AstraZeneca, Pfizer, and Bristol-Myers Squibb, and honoraria from Eli Lilly, Merck Sharp & Dohme, Roche, Pharmamar, Merck, AstraZeneca, Novartis, Servier, Amgen, Pfizer, Sanofi, Bayer, Bristol-Myers Squibb, Mirati, GlaxoSmithKline, Janssen, Takeda, and Mirati. Dr. Lopez-Rios has received research funding from Eli Lilly, AstraZeneca, Roche, Pfizer, and ThermoFisher Scientific, and honoraria from Abbvie, Astellas, AstraZeneca, Bayer, Bristol-Myers Squibb, Daiichi-Sankyo, Janssen, Eli Lilly, Merck Sharp & Dohme, Merck, Pfizer, Roche, Sanofi, Takeda, and Thermo Fisher. The remaining authors declare no conflict of interest.
